# At-Sea Associations in Foraging Little Penguins

**DOI:** 10.1371/journal.pone.0105065

**Published:** 2014-08-13

**Authors:** Maud Berlincourt, John P. Y. Arnould

**Affiliations:** School of Life and Environmental Sciences, Deakin University, Burwood, Victoria, Australia; Hawaii Pacific University, United States of America

## Abstract

Prey distribution, patch size, and the presence of conspecifics are important factors influencing a predator’s feeding tactics, including the decision to feed individually or socially. Little is known about group behaviour in seabirds as they spend most of their lives in the marine environment where it is difficult to observe their foraging activities. In this study, we report on at-sea foraging associations of little penguins (*Eudyptula minor*) during the breeding season. Individuals could be categorised as (1) not associating; (2) associating when departing from and/or returning to the colony; or (3) at sea when travelling, diving or performing synchronised dives. Out of 84 separate foraging tracks, 58 (69.0%) involved associations with conspecifics. Furthermore, in a total of 39 (46.4%), individuals were found to dive during association and in 32 (38.1%), individuals were found to exhibit synchronous diving. These behaviours suggest little penguins forage in groups, could synchronise their underwater movements and potentially cooperate to concentrate their small schooling prey.

## Introduction

Locating and exploiting resources is a constant challenge for animals. It is a time when cooperative strategies can greatly improve the probability of being successful. Breeding seabirds are central place foragers [1] and show specific adaptations to the difficulties encountered in finding food within the sparseness and patchiness of the marine environment they exploit [2]. In such an environment, group foraging could benefit individuals by increasing their efficiency at finding resources, consequently increasing their feeding rate in comparison to solitary foragers, and may also assist in the acquisition of food by concentrating small prey or by facilitating the capture of large prey [3].

Penguins consume small schooling fish, crustaceans and cephalopods [4] and previous studies have inferred group foraging behaviour from observations of individuals aggregating at the surface [5]–[7] or with the use of animal-borne camera loggers [8]–[10]. Recent bio-logging studies have documented synchrony between individuals in the departure from colonies, overlap in general foraging areas or synchronous underwater activity [11]–[13]. Although it has been shown that penguins can display group foraging behaviour, the degree of interaction between individuals is uncertain and little is known about how often penguins do associate when not diving. No studies have yet concurrently used depth recorders and GPS data loggers for a whole foraging trip to examine fine-scale spatial overlap in time and coordinated diving behaviour indicative of group foraging.

The little penguin (*Eudyptula minor*) is the smallest of all penguin species and is found exclusively in southern Australia, New Zealand and the Chatham Islands [14]. Breeding colonies are located on coastal mainland sites and islands where they are known to depart from, and return to, the breeding colony in groups [15]. However, whether individuals intentionally forage together at sea is not known in this (or other) penguin species. This study, therefore, investigated the degree of associations between individuals at sea and whether such associations were linked to foraging activity. In addition, as little penguins exhibit sexual size-dimorphism [16], with males diving longer and deeper than females [17] and potentially foraging in different zones, the effect of sex on at-sea associations was tested. Furthermore, because breeding colonies could serve as information centres where unrelated individuals transfer information on the location of food resources [18], we examined whether birds which nested in close proximity travelled together and/or foraged together.

## Materials and Methods

### Ethics Statement

The ethical guidelines of Deakin University Animal Ethics Committee and Animal Welfare Committee were followed during this present study. The protocol was approved by Deakin University Animal Ethics Committee and Animal Welfare Committee (Permit No. 10004786). The project was conducted in accordance with the regulations of Department of Sustainability and Environment (DSE) (Permit No. 10005531). London Bridge is part of the Port Campbell National Park and was access under permit from Parks Victoria.

### Study site and animal handling

The study was conducted at the London Bridge breeding colony (38°37′19″S, 142°55′57″E), south-eastern Australia, during the chick-rearing period in 2011/12 and 2012/13. This small mainland colony hosted 68 and 70 active nests in the first and second study periods, respectively. Chick-rearing lasts for 8–10 weeks and consists of two stages: guard stage (2 weeks) where adults make one-day foraging trips while the partner tends to the chicks; and post guard stage (6 to 8 weeks) where adults leave chicks unattended to forage at sea [19]. Breeding adults were instrumented with a GPS data logger (IgotU120, Mobile Action Technology, 44.5×28.5×13 mm), packaged in heat-shrink tubing, programmed to sample location (±10 m) every 2 min. In addition, individuals were deployed with a time-depth recorder (TDR LAT1500, Lotek Wireless Inc, 35×8×8 mm) to measure depth (±0.01 m) every 4 s. Adults were captured and instrumented at their nest with procedures lasting <10 min. Prior to deployment, morphometric measurements (mass and flipper length) were taken using a spring balance (±5 g) and Vernier calipers (±1 mm). Both individuals in each nest were known from previous studies (identified by passive induction transponders, PIT tags, [15]) and their sex had been determined from bill depth using a discriminant function [16]. Devices were attached with black waterproof Tesa tape to feathers on the dorsal midline. Together the devices weighed <3% of body mass and were <0.03% of body cross sectional surface area and, thus, would have had negligible hydrodynamic drag effects on the animals. Birds were recaptured and devices removed after returning from one foraging trip to sea. In addition, to investigate the potential influence of nesting proximity on associations, the distance (m) between studied nests was determined with a tape measure.

### Data Analyses

All analyses were conducted in the R statistical environment [20]. The *trip* package [21] was used to summarize animal track data. Locations with speed >2 m·s^−1^ were filtered [17], [22]. On average, the speed filtering removed 7.1% (range: 0–22.2%) of recorded locations during individual foraging trips. Analyses were performed on complete foraging trips, defined as the time between when individuals departed from, and when they returned to, the colony.

The *diveMove* package [23] was used to process and analyse TDR data. Following zero-offset correction and setting a minimum dive depth of 1 m, a sequential differences analysis [24] was used to search for bout-ending criterion and determine whether successive dives were part of the same bout. For each foraging trip, the average dive and post dive duration, dives per bout and bout duration were calculated. GPS tracking data were then linearly interpolated to estimate a location for each dive. Horizontal distance travelled during each dive bout was estimated from the linearly interpolated GPS tracking data.

To analyse inter-individual associations at sea, interpolated tracks and diving datasets were analysed using a custom-written script in Eonfusion (Myriax Pty. Ltd., Hobart, Australia). An individual track was analysed to see if another individual was present within a fixed radius at any time during the trip. The sum total time of associations between individuals was calculated for each trip. Little penguins often congregate at the water surface close to the colony (“rafting”) before emerging after sunset [14]. Tracks were visually examined and if two or more individuals were “rafting” together, these track segments were excluded from further analyses. In order to investigate possible factors affecting time spent in association, the effect of sex was tested using a non-parametric test and the correlation between nest proximity and time spent in association was tested using Mantel test [25]. The Mantel test evaluates correlations between two distance matrices of the same size. The Mantel statistic can be normalized such that it behaves like a correlation coefficient (*r*) varying from −1 to 1. The significance of the correlation between the two distance matrices was tested using a permutation test. Furthermore, the diving behaviour of associated individuals was examined. Dives statistics (number of dives, time of dive, dive duration, post-dive duration and dive depth) were calculated for each individual.

## Results

A total of 84 foraging trip tracks (32 female, 31 male) were recorded (see Appendix S1 and S2). The duration of foraging trips was 14.8±4.1 h during which individuals travelled 41.2±18.7 km. To determine whether instrumented individuals associated, a zone around the foraging track of 500 m radius was created. This radius was chosen based on the average horizontal distance travelled per dive bout (0.99±0.08 km). The mean dive bout duration (12.4±7.9 min) was then used as a threshold (minimum unit period) to define association between individuals. On average, 3 individuals (range: 2–7) were instrumented simultaneously and departed to sea on the same day (Table 1), which represented on average 9% (range: 5.1–20.8) of the total number of foraging adults raising chicks at the time of instrumentation. On average, 87.5% (range: 0–100) of simultaneously instrumented individuals were found to associate during their foraging trip (Table 1).

**Table 1:** Summary of individuals observed to associate and total number of individuals foraging at the time of instrumentation.

Date ofdeployment	Individualsinstrumented	Individuals associating for atleast one “dive boutduration” unit	Proportion of instrumentedindividuals observedto associate (%)	Number of individualsat sea foraging
01/10/2011	3	3	100	46
03/10/2011	5	5	100	46
04/10/2011	7	7	100	43
18/10/2011	3	3	100	59
20/08/2012	5	3	60	24
22/08/2012	5	5	100	24
23/08/2012	3	2	66.7	24
25/09/2012	3	2	66.7	39
26/09/2012	2	0	0	39
27/09/2012	2	2	100	39
30/10/2012	4	3	75	39
31/10/2012	4	3	75	39
20/11/2012	3	3	100	35
21/11/2012	2	2	100	35
22/11/2012	2	0	0	35
02/12/2012	3	3	100	35
03/12/2012	2	0	0	35
12/12/2012	4	2	50	22
13/12/2012	4	3	75	22
18/01/2013	3	2	66.7	32
19/01/2013	3	3	100	32
21/01/2013	2	2	100	32
Median[range]	3 [2]–[7]	3 [0–7]	87.5 [0–100]	35 [22–59]

The total number of adults foraging was calculated from the number of breeding pairs raising chicks in the colony, see Materials and Methods for more details.

Tracked individuals could be classified into three groups: (1) individuals that did not associate at all; (2) individuals that associated with conspecifics only during outward/inward commuting; and (3) individuals that, irrespective of whether they left the colony together or not, associated at sea. Within group 3, different degrees of associations were found between individuals: (a) individuals that associated when travelling; (b) individuals that associated when travelling and diving asynchronously within the same area; and (c) individuals performing synchronous diving (a pair was considered to exhibit synchronous diving behaviour when the two individuals initiated a dive within 4 s of each other) (Table 2). Out of the 84 separate tracks, 58 (69%) were classified into groups 2 or 3 with the highest number of individuals corresponding to groups 3b and 3c (Table 2).

**Table 2:** Different types of associations found in instrumented little penguins.

At-sea associations	Number ofindividuals	Proportion of timespenttravellingwhile associated (%)	Time spent asynchronouslydiving while associated (%)	Time spent divingsynchronously (%)	Proportion of the foragingtrip duration spent inassociation (%)
1. No association	26	-	-	-	-
2. Outward/inwardcommuting	10	4.1 [1.3–6.5]	-	-	4.1 [1.3–6.5]
3. At-sea:					
a. Surfacetravelling	6	4.1 [1.3–5.3]	-	-	4.1 [1.3–5.3]
b. Travelling anddiving	39	2.2 [0.1–13.6]	1.9 [0.1–10.3]	-	3.8 [0.2–20.6]
c. Synchronousdiving	32	7.2 [0.8–32.6]	1.6 [0.1–10.3]	6.3 [0.3–73.3]	20.9 [4.2–89.7]

The proportion of time spent travelling while associated is the proportion of time an individual spent travelling at the surface only relative to its foraging trip duration. The proportion of time spent diving while associated is the time an individual spent diving asynchronously relative to its foraging trip duration. Median values are given with range in parentheses.

**Figure 1:**
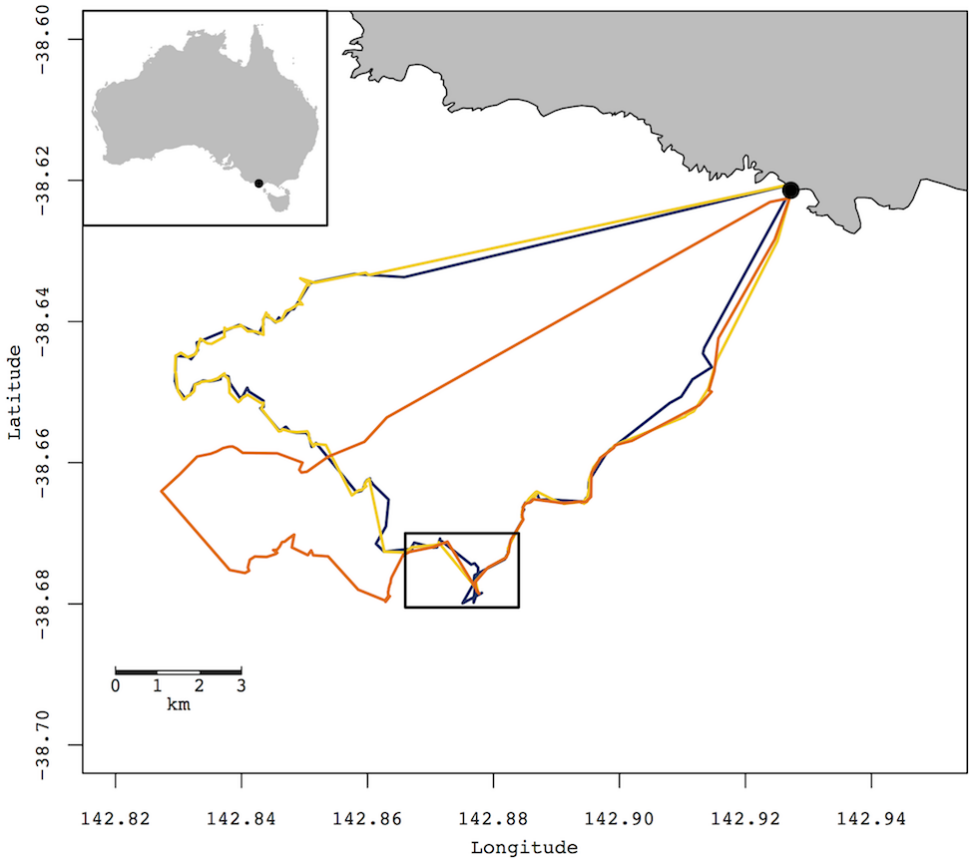
Foraging trips of three little penguins tracked with GPS. Example of GPS tracks from three foraging adult little penguins at the London Bridge breeding colony (black dot). The rectangle represents dive events location showed in Figure 2.

**Figure 2:**
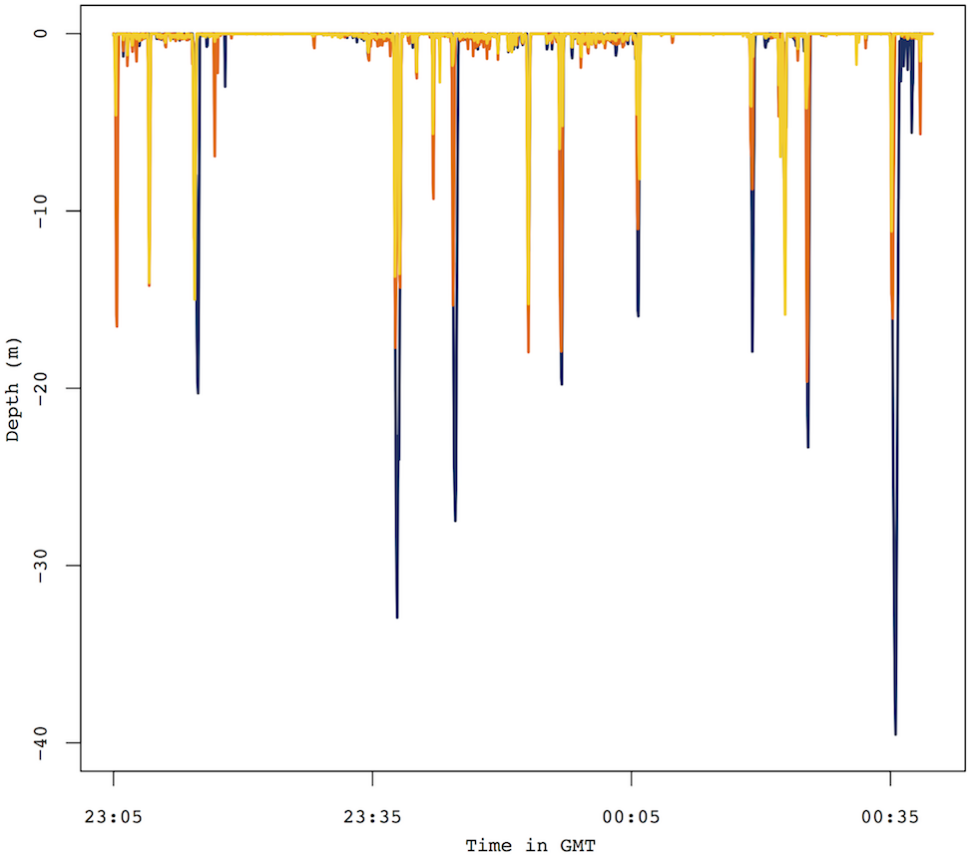
Dives profiles of three little penguins illustrating synchronous diving behaviour. Representative example of synchronous dives by three little penguins during a portion of their foraging trips.

On average, individuals that associated during the outgoing and/or incoming stages of the foraging trip were found to interact for 4.1% of their foraging trip. In contrast, individuals in group 3c spent up to 89.7% (average 20.9%) of their time at sea in associations (Table 2; Figure 1). Birds that were diving synchronously spent on average 6.3% of their foraging trip doing so for an average of 5 (range: 1–70) synchronized dives (Table 2 and Table 3; Figure 2). While diving, the distance association at the surface between 2 individuals was on average 92.1 m (range: 34.1–495.3) (Table 3). Sex did not influence time spent in association with a conspecific (Mann-Whitney’s U test, U = 939, *p* = 0.453). Distances between all pairs of nests were measured (55.4±61.2 m) (see Appendix S3). Time spent in association did not appear to be correlated to nesting proximity (*r* = −0.02, *p* = 0.339).

**Table 3:** Diving parameters of instrumented little penguins pairs that associated while synchronously diving.

Pair	Durationof association (min)	Numberof dives	Dive duration (s)	Depth (m)	Postdive duration (s)	Surface distance (m)
ID35/ID56	4	1	4	1.1	-	290.1
ID48/ID49	4.7	4	18 [4–44]	2.8 [1–13.7]	50 [8–152]	329.1
ID29/ID41	4.9	5	14 [4]–[28]	2.9 [1.6–8]	72 [4–108]	48.3 [10.2–355.8]
ID28/ID49	12.2	3	8 [8]–[20]	4.3 [3.3–9.6]	4 [4]–[16]	495.3
ID5/ID6	12.5	6	36 [28–48]	10.6 [7.9–14.4]	16 [8]–[28]	63.0 [14.2–184.7]
ID1/ID2	18.5	2	28 [24]–[32]	4.5 [1–7.9]	20 [8]–[32]	375.2
ID11/ID15	21.3	4	28 [20–52]	6.2 [1.2–15.6]	38 [20–116]	317.2
ID24/ID82	27.9	5	24 [4–44]	5.4 [1–8.1]	20 [4–148]	112.6 [72.3–338.1]
ID14/ID16	32.7	4	28 [12]–[32]	9.2 [3.4–12.1]	26 [4–48]	344.7 [287.3–402.2]
ID13/ID19	34.5	4	40 [8–56]	12.9 [2.1–18.5]	28 [24–128]	79.4 [53.7–105.1]
ID63/ID65	83.7	20	28 [12–48]	8.0 [1–12.4]	24 [12–116]	218.9 [44.5–480.9]
ID66/ID69	91.2	9	8 [4–48]	3.6 [1.2–17.8]	44 [4–160]	141.1 [34.9–264]
ID15/ID17	121.1	3	42 [24–60]	16.9 [8.6–22.1]	34 [16–68]	203.2 [194.6–471.5]
ID19/ID20	135.7	8	38 [4–48]	11.6 [1.2–15.2]	32 [8–128]	49.3 [20.8–132.7]
ID29/ID40	178.8	11	10 [4–56]	2.3 [1.1–4]	62 [4–160]	97.1 [11.2–371.7]
ID37/ID57	199.5	3	10 [4]–[16]	2.8 [1.6–8.6]	46 [4–76]	94.9 [24.7–159.9]
ID50/ID52	248.9	57	32 [4–96]	8.2 [1.1–16.8]	20 [4–168]	201.8 [7.4–462.9]
ID6/ID8	276.2	70	40 [4–64]	11.1 [1.1–26.5]	20 [4–80]	39.4 [2.6–405.3]
ID12/ID14	357.3	30	44 [20–64]	12.1 [1.1–21.7]	20 [8–80]	34.1 [1.8–199.8
ID40/ID41	479.5	68	12 [4–48]	2.4 [1–24.9]	30 [4–176]	119.1 [5.9–429.8]
Median[range]	59.1 [4–479.5]	5 [1–70]	28 [4–44]	8.1 [1.1–16.9]	24 [4–176]	92.1 [34.1–495.3]

The number of dives is the total dives synchronously performed by a pair. The surface distance is the distance between two individuals at the time of association. Median values are given with range in parentheses.

## Discussion

The results indicate that in little penguins, even while a relatively small proportion of the colony was concurrently tracked, a large proportion of instrumented individuals associated with a conspecific during a foraging trip. This suggests that little penguins are unlikely to forage individually and that group association occurs in this species, as has been previously described for other penguin species [6], [7], [26]. However, while previous studies inferred group foraging behaviour from observations of individuals at the surface or from information on underwater activity recorded with time-depth recorders alone [10]–[13], the present study emphasised the existence of different degrees of associations between birds. Some individuals departed from the colony together and separated and/or came back towards the colony and merged with others before rafting while waiting until sunset to go back to the colony. Others spent time at sea together, regardless of whether they had left the colony together or not.

The proportion of the foraging trip duration spent in association was higher when the individuals were diving than travelling. This suggests individuals occasionally encountered and fed on a common prey patch. Some individuals synchronised their underwater movements during almost the entire foraging trip. In both cases, these observations suggest individuals were involved in cooperative foraging.

The benefits of group hunting are multiple. It reduces an individual’s risk of being preyed upon [27] and it can provide cues to locate food in an environment where the resources are patchily and unpredictably distributed which may enhance foraging efficiency [28]. On one hand, penguins might be forming groups in order to split school prey formation. Indeed, fragmentation reduces dilution, which is one of the greatest strength behind the evolution of schooling behaviour [29]. Therefore, penguins might need to disrupt school cohesion to capture prey. On the other hand, they possibly need the prey not to disperse too much. In fact, penguins may need to herd prey because, if they disperse, hunting would not be cost-efficient anymore. Therefore, prey might need to be kept in small group formation. It has been suggested that penguins are more likely to be successful if operating in small groups when hunting small aggregations of prey [30]. Furthermore, school-response patterns differ with school size [31]. As consumers of small schooling prey (e.g. anchovies or pilchards, [14]), little penguins could adapt their tactics according to the size of the school. Penguins could benefit from cooperative hunting to maintain a certain degree of disorder and/or aggregation that facilitates prey capture. Conversely, group hunting can also increase competition for resources when prey availability is limited and, therefore, it can become costly for an individual to associate with conspecifics. Differences in the degree of association observed between individuals in the present study could, therefore, be reflective of differences in prey distribution patterns or differing characteristics in prey [32].

Nesting proximity did not appear to influence group formation. This result is surprising because little penguins emerge from their burrows at approximately the same time and walk along communal tracks in the dunes to the water where they congregate before departing from the colony [33]. As sampling for this study was mostly conducted when parents alternated foraging and brooding, it was not possible to investigate whether nesting pairs forage together. While some adults were in post-guard stage at the time of the instrumentation, no pair was tracked at the same time. Further study during the post guard stage when both parents forage at the same time could potentially investigate whether pairs forage together. While small sample size (only a few individuals tracked at the same time) prevented determination of whether sex influenced group formation, the duration of associations was not related to sex.

This study has shown that little penguins associate with conspecifics while foraging at sea and can adjust underwater hunting activity to that of other individuals; this could suggest cooperative foraging or opportunistic behaviour. Although this study only followed few individuals at the same time for a single foraging trip (due to limitations in the field), the findings indicate that individuals preferentially spent time together. However, future studies should incorporate larger groups and over numerous trips in order to investigate whether individuals maintain associations over multiple trips and/or develop regular associations with particular individuals (e.g. genetically related).

## Supporting Information

Appendix S1 **Summary information of individuals instrumented during the two breeding seasons.**
(PDF)

Appendix S2 **Linearly interpolated GPS tracking data.**
(CSV)

Appendix S3 **Nest distances measured between all individuals instrumented.**
(PDF)

## References

[1] Orians GH, Pearson NE (1979) On the theory of central place foraging. In: Horn DJ, Stairs GR, Mitchell RD, editors. *Analysis of ecological systems*. Columbus: Ohio State University Press. 155–177.

[2] Ashmole NP (1971) Seabird ecology and the marine environment. In: Farner DS, King JR, editors. *Avian Biology*. New York, N.Y., U.S.A: Academic Press. 223–286.

[3] Clark CW, Mangel M, (1986) The evolutionary advantages of group foraging. Theor Popul Biol, 30, 45–75. 10.1016/0040-5809(86)90024-9

[4] Marchant S, Higgins PJ (1990) *Handbook of Australian, New Zealand and Antarctic birds*. Melbourne: Oxford University Press.

[5] Wilson RP, Wilson M (1990) Foraging ecology of breeding *Spheniscus* penguins. In: Davis L, Darby JT, editors. *Penguin biology*. London: Academic Press. 181–206.

[6] Ainley D (1972) Flocking in Adélie penguins. Ibis, 114, 388–390. 10.1111/j.1474-919X.1972.tb00836.x

[7] Siegfried W, Frost P, Kinahan J, Cooper J (1975) Social behaviour of Jackass Penguins at sea. Zoo Afr, 10, 87–100.

[8] Takahashi A, Kokubun N, Mori Y, Shin H-C, (2008) Krill-feeding behaviour of gentoo penguins as shown by animal-borne camera loggers. Polar Biology, 31, 1291–1294. 10.1007/s00300-008-0502-4

[9] Kokubun N, Kim J-H, Takahashi A, (2013) Proximity of krill and salps in an Antarctic coastal ecosystem: evidence from penguin-mounted cameras. Polar Biology, 36, 1857–1864. 10.1007/s00300-013-1400-y

[10] Takahashi A, Sato K, Naito Y, Dunn MJ, Trathan PN, et al (2004) Penguin-mounted cameras glimpse underwater group behaviour. Proc Biol Sci, 271 Suppl 5, 281–282. 10.1098/rsbl.2004.0182 PMC1810073 15503994

[11] Tremblay Y, Cherel Y (1999) Synchronous underwater foraging behavior in penguins. The Condor, 101, 179–185.

[12] Pütz K, Cherel Y (2005) The diving behaviour of brooding king penguins (*Aptenodytes patagonicus*) from the Falkland Islands: variation in dive profiles and synchronous underwater swimming provide new insights into their foraging strategies. Mar Biol, 147, 281–290. 10.1007/s00227-005-1577-x

[13] Takahashi A, Sato K, Nishikawa J, Watanuki Y, Naito Y (2004) Synchronous diving behavior of Adelie penguins. J Ethol, 22, 5–11. 10.1007/s10164-003-0111-1

[14] Stahel C, Gales R, Burrell J (1987) *Little penguin: Fairy penguins in Australia*. Kensington, NSW, Australia: New South Wales University Press.

[15] Daniel TA, Chiaradia A, Logan M, Quinn GP, Reina RD (2007) Synchronized group association in little penguins *Eudyptula minor* . Anim Behav, 74, 1241–1248. 10.1016/j.anbehav.2007.01.029

[16] Arnould J, Dann P, Cullen J (2004) Determining the sex of Little Penguins (*Eudyptula minor*) in northern Bass Strait using morphometric measurements. Emu, 104, 261–265. 10.1071/MU04035

[17] Hoskins AJ, Dann P, Ropert-Coudert Y, Kato A, Chiaradia A, et al (2008) Foraging behaviour and habitat selection of the little penguin *Eudyptula minor* during early chick rearing in Bass Strait, Australia. MEPS, 366, 293–303. 10.3354/meps07507

[18] Ward P, Zahavi A (1973) The importance of certain assemblages of birds as “information-centres” for food-finding. Ibis, 115, 517–534. 10.1111/j.1474-919X.1973.tb01990.x

[19] Reilly PN, Cullen JM (1981) The little penguin *Eudyptula minor* in Victoria II: Breeding. Emu, 81, 1–19. 10.1071/MU9810001

[20] R Developement Core Team. 2013 R: A language and environment for statistical computing. ISBN 3-900051-07-0. R Foundation for Statistical Computing. Vienna, Austria. Available: http://www.R-project.org.

[21] Sumner MD. 2012 trip: Spatial analysis of animal track data. R package version 1.1-12. Available: http://CRAN.R-project.org/package=trip.

[22] McConnell B, Chambers C, Fedak M (1992) Foraging ecology of southern elephant seals in relation to the bathymetry and productivity of the Southern Ocean. Antarct Sci, 4, 393–398.

[23] Luque SP (2007) Diving Behaviour Analysis in R. R News. 7(3): 8–14.

[24] Mori Y, Yoda K, Sato K (2001) Defining dive bouts using a sequential differences analysis. Behaviour, 138, 1451–1466.

[25] Mantel N (1967) The detection of disease clustering and a generalized regression approach. Cancer Res, 27, 209–220. 6018555

[26] Davis LS, Darby JT (1990) *Penguin biology*. Academic Press.

[27] Lima SL, Dill LM (1990) Behavioral decisions made under the risk of predation: a review and prospectus. Can J Zool, 68, 619–640. 10.1139/z90-092

[28] Grand TC, Dill LM (1999) The effect of group size on the foraging behaviour of juvenile coho salmon: reduction of predation risk or increased competition? Anim Behav, 58, 443–451. 10.1006/anbe.1999.1174 10458896

[29] Pitcher T, Parrish J (1993) Functions of shoaling behaviour in teleosts. In: Pitcher T, editor. *Behaviour of Teleost Fishes*. London: Chapman & Hall. 363–439.

[30] Wilson RP, Wilson MPT, McQuaid L (1986) Group size in foraging African penguins (*Spheniscus demersus*). Ethology, 72, 338–341. 10.1111/j.1439-0310.1986.tb00634.x

[31] Vabø R, Nøttestad L, (1997) An individual based model of fish school reactions: predicting antipredator behaviour as observed in nature. Fisheries Oceanography, 6, 155–171. 10.1046/j.1365-2419.1997.00037.x

[32] Packer C, Ruttan L (1988) The evolution of cooperative hunting. Am Nat, 159–198.

[33] Chiaradia AF, Kerry KR, (1999) Daily nest attendance and breeding performance in the little penguin *Eudyptula minor* at Phillip Island, Australia. Mar Ornithol, 27, 13–20.

