# Supplementary material for: At-Sea Associations in Foraging Little Penguins
Source: PLoS One. 2014 Aug 13;9(8):e105065. doi: 10.1371/journal.pone.0105065 (PMC4132066; doi:10.1371/journal.pone.0105065)
Supplement: Appendix S1 — Summary information of individuals instrumented during the two breeding seasons. (PDF) [file pone.0105065.s001.pdf]

Stage: GS=guard stage / PGS=post guard stage

Year	ID	Sex	Stage	Nest
2011/2012	1	F	GS	20
2011/2012	2	M	GS	33
2011/2012	3	F	GS	41
2011/2012	4	M	GS	31
2011/2012	5	F	GS	1
2011/2012	6	M	GS	15
2011/2012	7	F	GS	14
2011/2012	8	F	GS	26
2011/2012	9	F	GS	45
2011/2012	10	M	GS	20
2011/2012	11	F	GS	33
2011/2012	12	M	GS	41
2011/2012	13	F	GS	31
2011/2012	14	F	GS	15
2011/2012	15	M	GS	14
2011/2012	16	M	GS	26
2011/2012	17	M	GS	45
2011/2012	18	F	PGS	21
2011/2012	19	M	PGS	64
2011/2012	20	F	PGS	53
2011/2012	21	M	PGS	21
2012/2013	22	F	GS	4
2012/2013	23	F	PGS	20
2012/2013	24	F	PGS	20
2012/2013	25	M	PGS	33
2012/2013	26	M	GS	15
2012/2013	27	M	GS	15
2012/2013	28	F	GS	45
2012/2013	29	M	GS	4
2012/2013	30	M	PGS	20
2012/2013	31	M	PGS	20
2012/2013	32	F	GS	33
2012/2013	33	F	GS	15
2012/2013	34	F	GS	15
2012/2013	35	M	PGS	26
2012/2013	36	M	GS	45
2012/2013	37	F	PGS	23
2012/2013	38	F	GS	3
2012/2013	39	F	GS	3
2012/2013	40	F	GS	35
2012/2013	41	F	GS	59
2012/2013	42	F	GS	59
2012/2013	43	M	GS	59

2012/2013	44	M	GS	59
2012/2013	45	M	GS	3
2012/2013	46	M	GS	8
2012/2013	47	M	GS	8
2012/2013	48	M	GS	5
2012/2013	49	F	GS	27
2012/2013	50	F	GS	8
2012/2013	51	F	GS	8
2012/2013	52	F	GS	5
2012/2013	53	F	GS	5
2012/2013	54	F	PGS	87
2012/2013	55	F	PGS	87
2012/2013	56	M	PGS	87
2012/2013	57	M	PGS	23
2012/2013	58	M	GS	28
2012/2013	59	F	GS	57
2012/2013	60	M	PGS	56
2012/2013	61	F	PGS	25
2012/2013	62	F	PGS	56
2012/2013	63	M	GS	57
2012/2013	64	F	GS	28
2012/2013	65	M	GS	83
2012/2013	66	M	PGS	50
2012/2013	67	M	PGS	39
2012/2013	68	F	PGS	76
2012/2013	69	F	PGS	51
2012/2013	70	F	PGS	7
2012/2013	71	M	PGS	7
2012/2013	72	M	PGS	76
2012/2013	73	F	GS	38
2012/2013	74	M	GS	38
2012/2013	75	M	GS	61
2012/2013	76	F	GS	13
2012/2013	77	F	GS	61
2012/2013	78	M	GS	13
2012/2013	79	M	GS	22
2012/2013	80	F	GS	22
2012/2013	81	F	GS	66
2012/2013	82	M	GS	66
2012/2013	83	M	GS	6
2012/2013	84	F	GS	6
