# Supplementary material for: At-Sea Associations in Foraging Little Penguins
Source: PLoS One. 2014 Aug 13;9(8):e105065. doi: 10.1371/journal.pone.0105065 (PMC4132066; doi:10.1371/journal.pone.0105065)
Supplement: Appendix S3 — Nest distances measured between all individuals instrumented. (PDF) [file pone.0105065.s003.pdf]

ID	distance (m)
ID34/ID22	60
ID2/ID1	17
ID3/ID1	51
ID82/ID24	13
ID83/ID24	4
ID3/ID2	39
ID1/ID2	17
ID55/ID25	10
ID1/ID3	51
ID2/ID3	39
ID8/ID5	38
ID9/ID5	177
ID6/ID5	15
ID5/ID6	15
ID8/ID6	32
ID46/ID6	20
ID49/ID26	28
ID8/ID7	26
ID9/ID7	170
ID6/ID8	32
ID5/ID8	38
ID7/ID8	26
ID7/ID9	170
ID5/ID9	177
ID48/ID28	162
ID49/ID28	134
ID32/ID31	17
ID12/ID11	39
ID17/ID11	135
ID15/ID11	33
ID14/ID11	40
ID13/ID11	44
ID31/ID32	17
ID14/ID12	71
ID11/ID12	39
ID16/ID12	40
ID17/ID12	123
ID15/ID12	66
ID11/ID13	44
ID17/ID13	150
ID16/ID14	32
ID11/ID14	40
ID15/ID14	2
ID12/ID14	71
ID17/ID14	172

ID22/ID34	60
ID73/ID34	57
ID11/ID15	33
ID12/ID15	66
ID17/ID15	170
ID14/ID15	2
ID12/ID16	40
ID14/ID16	32
ID56/ID35	4
ID15/ID17	170
ID14/ID17	172
ID12/ID17	123
ID13/ID17	150
ID11/ID17	135
ID20/ID18	208
ID19/ID18	15
ID18/ID19	15
ID20/ID19	230
ID18/ID20	208
ID19/ID20	230
ID57/ID37	0
ID41/ID38	63
ID40/ID38	30
ID40/ID38	30
ID29/ID38	3
ID77/ID39	130
ID51/ID39	7
ID29/ID40	30
ID38/ID40	30
ID41/ID40	15
ID38/ID40	30
ID38/ID41	63
ID29/ID41	60
ID40/ID41	15
ID48/ID46	3
ID49/ID46	14
ID26/ID46	20
ID46/ID48	3
ID49/ID48	14
ID28/ID48	162
ID46/ID49	14
ID26/ID49	28
ID28/ID49	134
ID48/ID49	14
ID52/ID50	3
ID39/ID51	7

ID77/ID51	69
ID50/ID52	3
ID25/ID55	10
ID35/ID56	4
ID37/ID57	0
ID60/ID59	1
ID59/ID60	1
ID61/ID60	2
ID60/ID61	2
ID64/ID62	1
ID63/ID62	1
ID65/ID62	29
ID65/ID63	30
ID64/ID63	3
ID62/ID63	1
ID62/ID64	1
ID65/ID64	25
ID63/ID64	3
ID63/ID65	30
ID62/ID65	29
ID64/ID65	25
ID67/ID66	177
ID69/ID66	180
ID69/ID67	180
ID66/ID67	177
ID67/ID69	180
ID66/ID69	180
ID72/ID71	5
ID71/ID72	5
ID34/ID73	57
ID22/ID73	60
ID76/ID75	145
ID75/ID76	145
ID51/ID77	69
ID24/ID82	13
ID83/ID82	14
ID24/ID83	4
ID82/ID83	14
